# Prevalence and predictors of loneliness among university students: A cross-sectional study

**DOI:** 10.1371/journal.pmen.0000375

**Published:** 2025-07-14

**Authors:** Abu Sayed Md. Al Mamun, Md. Lazu Islam, Mahir Hossain Labib, Farhana Hasan, Md. Ripter Hossian, Md. Nurul Islam, Md. Golam Hossain

**Affiliations:** Department of Statistics, Universiry of Rajshahi, Bangladesh; PLOS: Public Library of Science, UNITED KINGDOM OF GREAT BRITAIN AND NORTHERN IRELAND

## Abstract

Loneliness is recognized as a significant public health concern among university students, with potential implications for their physical and mental well-being, as well as their academic performance. However, the research on loneliness among university students in Bangladesh after COVID-19 pandemic is not available. This study aimed to find out how often university students in Bangladesh feel lonely and what factors contribute to it. The cross-sectional study was conducted from January 01 to February 29, 2024. Data was collected from 400 Rajshahi university students; they were selected by two-stage random sampling. We used the logistic regression model to find the predictors of loneliness, considering a p-value of less 0.05 as statistically significant. Data were analyzed using the SPSS software, version 22.0. This study raveled that more than 22% of university students were suffering from loneliness. The logistic regression model demonstrated that (i) younger (age ≤ 21years) [AOR = 1.83, 95% CI: 1.05-3.17; p < 0.05], (ii) both underweight [AOR = 2.32, 95% CI: 1.26-4.28; p < 0.01] and overweight [AOR = 2.74, 95%CI:1.27-5.92], (iii) living in nuclear family [AOR = 2.02, 95%CI:1.05-3.90] and (iv) students coming from poor family (family income<15,000 BDT) [AOR = 2.10, 95%CI: 1.05-4.17; p < 0.05] were more in risk to experience loneliness compared to their counterparts. The study finds that a remarkable number of university students are suffering from loneliness and has identified some modifiable predictors of the problem. The predictors provide valuable insights for reducing loneliness among university students. It is urgent for university authorities and policymakers of the country to improve policy for reducing loneliness among adult students.

## Introduction

Loneliness among university students is a significant yet often overlooked issue, particularly during the transition to university life. Leaving behind familiar support systems, students struggle to form meaningful connections, increasing feelings of loneliness. Academic pressures and the social need to fit in amplify these feelings [1,2]. Surprisingly, increased digital communication has worsened loneliness, as online interactions fail to provide the deep connection needed to ease it [3,4]. The COVID-19 pandemic further heightened loneliness globally, with vulnerable populations, including young adults, experiencing elevated levels of isolation [5–7].

Loneliness significantly impacts mental health, causing hopelessness, sadness, and self-harm thoughts. It also disrupts sleep patterns and hinders social bonding. Moreover, loneliness correlates with unhealthy behaviors like smoking and drug use, increasing risks of poor quality of life and premature death [8–13]. Among university students, age influences loneliness, with younger students facing adjustment challenges and older students experiencing career-related stress despite social networks [14–16]. This study hypothesizes that age significantly contributes to loneliness (H_01_).

BMI also plays a role, as students with high or low BMI face stigma, body dissatisfaction, and mental health challenges, which limit social interactions and exacerbate loneliness [17–22]. Hence, the second hypothesis postulates that BMI significantly affects loneliness (H_02_). Family structure influences loneliness through social support and interaction differences. Joint families offer frequent emotional support, reducing loneliness, but may sometimes feel restrictive. Nuclear families encourage independence but may lack broader support systems [23–26]. Thus, family structure is hypothesized to play a significant role in loneliness (H_03_). Family income also impacts loneliness by affecting access to social resources. Low-income students face financial stress, social exclusion, and mental health issues, increasing loneliness. In contrast, higher-income students have better access to resources but may experience loneliness due to emotional neglect or academic pressure [27–31]. This leads to the hypothesis that family income significantly influences loneliness (H_04_).

Numerous studies have highlighted the strong links between loneliness, academic performance, and psychological well-being among university students. Poor academic outcomes can both stem from and contribute to feelings of isolation, while psychological stress, including depression and anxiety, has consistently been found to correlate with loneliness [32,33]. Globally, loneliness has been driven by urbanization, changing family dynamics, and social fragmentation, with COVID-19 exacerbating the issue through disrupted interactions [34]. In Bangladesh, extended family systems and community networks traditionally mitigated loneliness. However, the pandemic strained these systems due to mobility restrictions, economic downturns, and health anxieties [35]. Digital exclusion in rural and low-income areas worsened isolation, while societal stigma around mental health masked the true extent of loneliness.

Understanding loneliness among university students is crucial, particularly at Rajshahi University, the second-largest public university in Bangladesh, where few studies have explored its prevalence and risk factors [36–38]. This research aims to investigate the prevalence and factors influencing loneliness among students in this unique cultural and academic context.

## Methods

### Ethics statement

The present study was conducted in accordance with the Declaration of Helsinki and was approved by the Ethics Committee of Institute of Biological Sciences, University of Rajshahi, Bangladesh (Memo No. 110(16)/320/IAMEBBC/IBSc, dated June 05, 2022). Participants were explained about the objective of the study, and we obtained their written consent.

### Study design and setting

A cross-sectional survey was conducted from January 1 to February 29, 2024 at Rajshahi University, which is the second-largest university in Bangladesh. The study included all full-time students enrolled at the university. Exclusion criteria comprised students having serious illness and who did not provide informed consent for participation or incompletely responded to the questionnaire.

### Sample size determination

All students in Rajshahi University were considered as the population for this study, which included approximately 34,000 students. To determine the sample size, we used a formula: n = N/(1 + N d^2^) [39]. In this formula, n represents the required sample size, N is the population size (in this case, 34,000 students), d is the chosen marginal error (set at 0.05) and 95% confidence level was considered. The formula indicated that 396 samples were required for the study, however we initially considered 436 (10% absent rate).

### Sample selection procedure

This study used a two-stage random sampling method to select participants. Four residential Halls (two male and two female) were randomly selected (Lottery method) from 11 male and 6 female Halls in the first stage. In the next stage, 218 male and 218 female students were randomly chosen, with equal allocation from each selected Halls. All relevant information was gathered from the respective residential hall offices. Additionally, permission was obtained from the respective residential hall authorities.

### Data collection procedure

Data was collected using partially self-developed pre-tested structured questionnaire. The questionnaire consisted of general information of students, and the 20-item University of California, Los Angeles (UCLA) loneliness scale [40,41]. Before collecting data, we explained the study’s purpose to the chosen students and obtained their written consent. Unfortunately, 36 selected students did not agree to provide their information. Finally, we considered 400 students (200 male and 200 female) as a sample for the study. First of all, a pilot survey was conducted with 60 students (15% of the total sample) to observe whether there was any lack or drawback in the questionnaire. After proper modification, the questionnaire was finalized and made ready for data collection.

### UCLA loneliness scale

The 20-items UCLA Loneliness Scale Version 3 [40,41] were used to measure loneliness among university students. The UCLA Loneliness Scale was already utilized to identify loneliness among university students in Bangladesh [37,38]. We selected the UCLA Loneliness Scale for this study because it is one of the most widely used and validated tools for measuring loneliness. It has demonstrated strong psychometric properties, including high reliability and validity, across diverse populations and settings. Additionally, its simple structure and adaptability make it suitable for assessing loneliness in our target group. While other tools are available, the UCLA Loneliness Scale’s widespread use facilitates comparability with findings from previous studies. Each item was rated on a four-point Likert scale (1 = “never,” 2 = “rarely,” 3 = “sometimes,” and 4 = “often”). The total score ranged from 20 (if someone never felt lonely) to 80 (if someone often felt lonely). There was no specific cutoff score to define loneliness [40,42]. After reverse coding certain items, a total score below 60 (indicating loneliness was “never” or “rarely”) suggested the person felt normal [43]. A score of 60 or higher (indicating loneliness was “sometimes” or “often”) suggested greater feelings of loneliness [38].

### Major variables

#### Outcome variables.

Student’s loneliness was the outcome variable of this study. If a student’s UCLA Loneliness Scale score was 60 or above, he/she is considered to have loneliness symptoms (coded as 1); otherwise, if his/her score was below 60, he/she is considered as normal (coded as 0).

#### Independent variables.

Some socio-economic, demographic and behavioral factors were considered as possible predictors of loneliness. We followed previous studies with university students to select the possible predictors and their categories [8,10,37]. The independent variables were mentioned in Table 1.

**Table 1:** Background characteristics of participants.

Study Variables	Group, N(%)	Study Variables	Group, N(%)
Gender	Male, 200 (50.0)	Residence	Urban, 191 (47.8)
	Female, 200 (50.0)		Rural, 209 (52.2)
Age Group	≤ 21 Years, 113 (28.2)	Religion	Muslim, 333 (83.3)
	> 21 Years, 287 (71.8)		Others, 67 (16.7)
Academic Year	First and second year, 154 (38.5)	Education Level of Father	Illiterate and Primary, 111 (27.7)
	Third year and above, 246 (61.5)		Secondary and above, 289 (72.3)
BMI	Underweight, 74 (18.5)	Education Level of Mother	Illiterate and Primary, 146 (36.5)
	Normal weight, 281 (70.2)		Secondary and above, 254 (63.5)
	Overweight, 45 (11.3)	Monthly Family Income	BDT < 15000, 89 (22.3)
Occupation of Father	Service, 110 (27.4)		BDT 15000- BDT 29999, 136 (34.0)
	Business, 107 (26.8)		BDT ≥30000, 175 (43.7)
	Others, 183 (45.8)	Family Type	Nuclear, 290 (72.5)
Occupation of Mother	Housewife, 349 (87.3)		Joint, 110 (27.5)
	Others, 51 (12.7)		

### Statistical analysis

Descriptive statistics and graphical representation were used to investigate participants’ personal information, their parents’ socio-economic status, and their experiences of loneliness. To find the association between symptoms of loneliness and different socio-economic factors of respondents, Chi-square test was used. Finally, logistic regression analysis was used to find the significant factors of loneliness among University students. The variance inflation factors (VIF) was used to check the multicollinearity problems among independent variables of the model, and we found no multicollinearity problems if the VIF was between 0 and 1 [44]. The models’ fitness was tested using the Hosmer and Lemeshow test. Accuracy was assessed by sensitivity, specificity, predictive values, and the ROC curve. All data were analyzed using SPSS software, version 22.0 (IBM, Armonk, NY, USA).

## Results

Table 1 provides a comprehensive overview of the participant demographic characteristics. Gender distribution was balanced, with an equal split between males and females. The majority of participants were aged over 21 years, with a smaller proportion being aged 21 years or younger. Academic enrollment was fairly distributed, with a significant portion in their Third Year and above. BMI distribution indicated a majority of normal-weight individuals, with a notable proportion classified as underweight or overweight. Residential distribution showed a slight preference for rural areas. Regarding religion, the majority identified as Muslim, while a minority belonged to other religious affiliations. Among fathers, a larger portion had secondary education or higher, while a smaller portion had up to primary education. For mothers, the majority had secondary education or higher, with a smaller group having up to primary education. Fathers’ occupations were distributed across the service sector, business, and other fields. Most mothers were housewives, with a smaller group working in other occupations. The majority of families were nuclear, with a smaller portion being joint families. In terms of monthly family income, the largest group had higher incomes, followed by those with middle-range incomes, and the smallest group had the lowest incomes.

**Fig 1:**
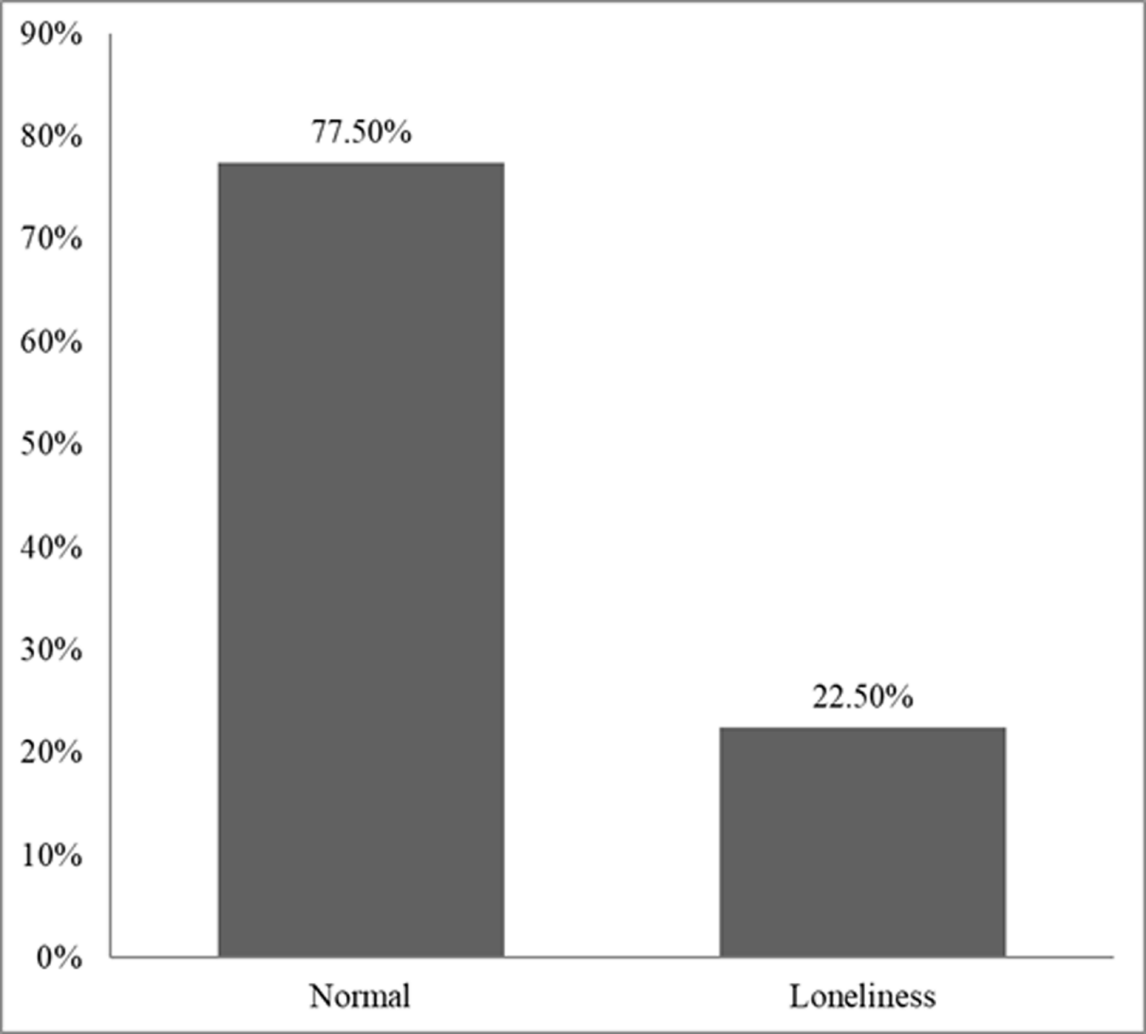
Prevalence of loneliness among university students.

Fig 1 illustrates the prevalence of loneliness among Rajshahi University students, based on a sample of 400 participants. It was observed that 22.5% of the students reported experiencing loneliness, while the rest of them were categorized as normal.

**Fig 2:**
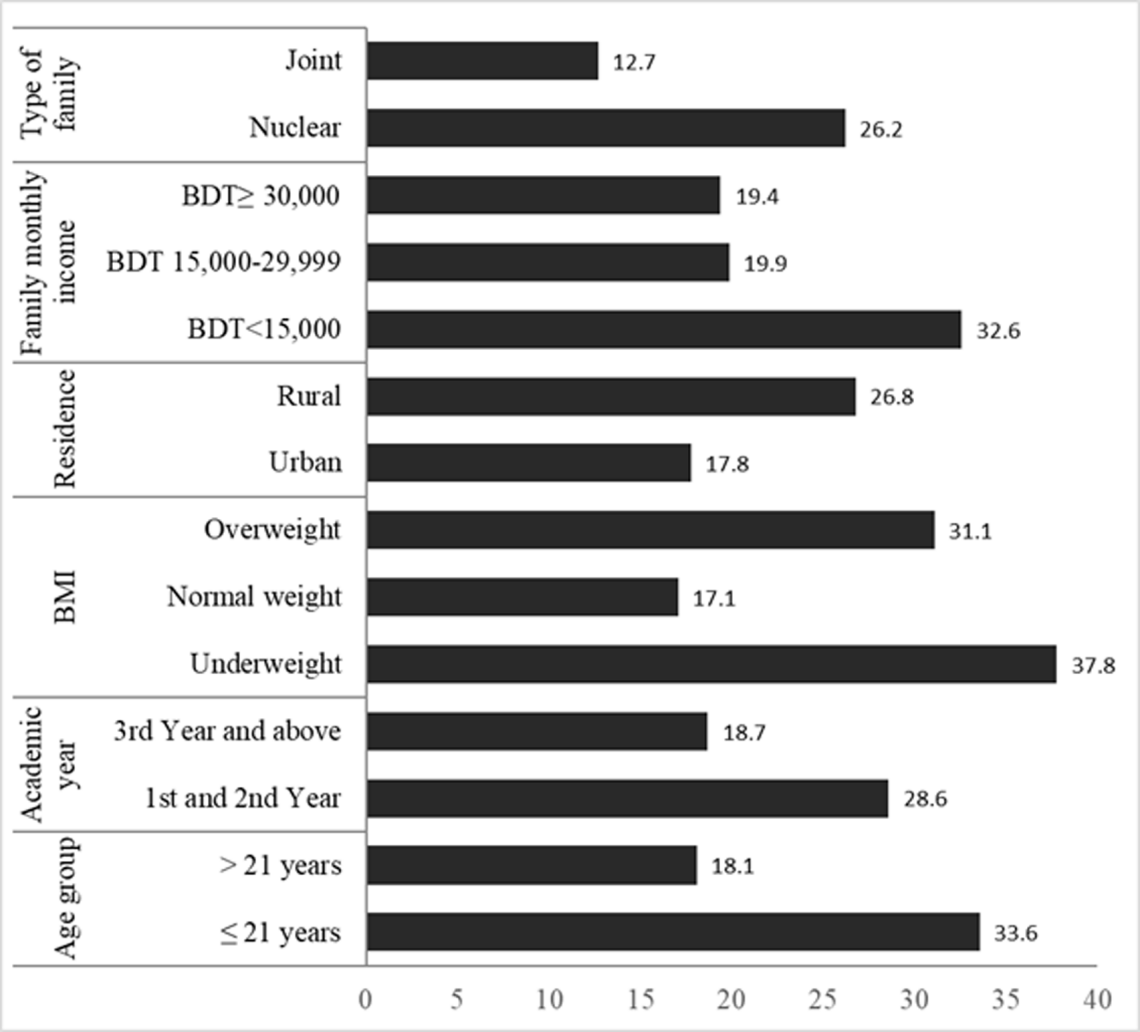
Prevalence of loneliness by different socio-economic factors of respondents.

Students aged 21 years or younger reported a higher prevalence of loneliness compared to those older than 21 years. Females reported higher loneliness compared to males. Students in their 1st and 2nd years experienced more loneliness than those in their 3rd year and above. Underweight students had the highest loneliness rates, followed by overweight students, and normal weight students. Students from rural areas felt lonelier compared to those from urban areas. Loneliness was more prevalent among students from families with a monthly income of less than BDT 15,000 compared to those with higher incomes. Students from nuclear families experienced more loneliness than those from joint families. Based on the Chi-square test, this study found that several factors such as age group (p < 0.01), academic years (p < 0.05), BMI (p < 0.01), residence (p < 0.05), family monthly income (p < 0.05), and type of family (p < 0.05) were statistical significant with loneliness, but the other selected factors such as gender, parents’ education, parents ‘occupations and religion did not show significance association with loneliness. Only the prevalence of loneliness of significant associated factors is shown in Fig 2.

**Fig 3:**
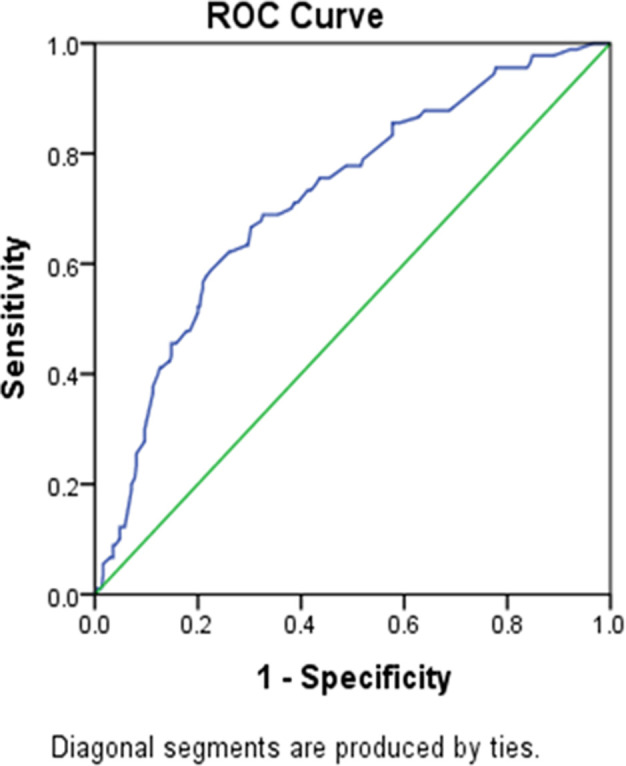
ROC for experience of loneliness among university students.

Table 2 summarizes the influence of various factors on respondents’ feelings of loneliness. Firstly, age played a role, as students aged 21 years or younger had higher odds of feeling lonely compared to those older than 21 years (AOR = 1.83, p = 0.030). Regarding BMI, both underweight and overweight students had significantly higher odds of experiencing loneliness compared to those with normal weight (Underweight: AOR = 2.32, p = 0.007; Overweight: AOR = 2.74, p = 0.010). Family type was another significant factor, with individuals from nuclear families having higher odds of feeling lonely compared to those from joint families (AOR = 2.02, p = 0.035). Additionally, family monthly income showed a significant effect, with individuals from households earning less than BDT 15,000 having higher odds of experiencing loneliness compared to those with incomes between BDT 15,000–29,999 (AOR = 2.10, p = 0.034). Hosmer and Lemeshow test showed that our selected model was good fitted (p > 0.05). The accuracy of the test was measured using the ROC curve. The area under the ROC curve was 0.719, which means that in 71.9% of the cases, the model correctly assigned a higher probability of experiencing loneliness to the subject who actually experienced it (Fig 3).

**Table 2:** Effect of various factors on respondents’ experience of loneliness.

Variables	Loneliness	p-value	AOR	95% CI for AOR	
	N (%)			Lower	Upper
**Age Group**					
≤ 21 years	38 (33.6)	0.030	1.83	1.05	3.17
> 21 years^R^	52 (18.1)				
**Academic Year**					
1^st^ & 2^nd^ Year	44 (28.6)	0.839	1.05	0.60	1.84
3^rd^ Year & above^R^	46 (18.7)				
**BMI**					
Underweight	28 (37.8)	0.007	2.32	1.26	4.28
Overweight	14 (31.1)	0.010	2.74	1.27	5.92
Normal Weight^R^	48 (17.1)				
**Residence**					
Rural	34 (17.8)	0.134	1.15	0.88	2.58
Urban^R^	56 (26.8)				
**Family Type**					
Nuclear	76 (26.2)	0.035	2.02	1.05	3.90
Joint^R^	14 (12.7)				
**Family Monthly Income**					
BDT < 15,000	29 (32.6)	0.034	2.10	1.05	4.17
BDT 15,000–29,999^R^	34 (19.4)	0.942	1.02	0.55	1.90
Hosmer and Lemeshow test	**χ**^**2**^ **– value = 2.76**			p-value = 0.95	

**N.B.:** B: Regression coefficients, CI: Confidence Interval, SE: Standard Error, BDT: Bangladeshi Currency, R: Reference category, AOR: Adjusted Odds Ratio.

## Discussion

In this study, 400 participants (200 male and 200 female) were selected to determine the prevalence and associated factors of loneliness among students..

The prevalence of loneliness was found to be 22.5%, which was surprisingly lower compared to previous studies conducted in Bangladesh [45,46]. This could be attributed to the timing of data collection, which occurred during the COVID-19 pandemic, a period when the prevalence of loneliness was at its peak [47–50]. The observed decline in loneliness is also supported by another study in Bangladesh [51], which may be explained by the gradual restoration of normalcy, increased social interactions, and reduced restrictions following the peak of the pandemic. Compared to Western countries, the prevalence in our study was lower, where rates often exceed 25% [52–54], likely due to higher levels of individualism in Western societies than in Bangladesh [55].

Another contributing factor to this discrepancy in the prevalence of loneliness was the lack of an identified cutoff point and Likert scale that defines loneliness on the UCLA loneliness scale [40,42]. However, the prevalence rate coincided with another study conducted among university students in medical science in Iran before COVID-19 [2].

Findings from this study revealed that gender did not significantly affect loneliness among university students. This finding was consistent with other researchers [36,56]. It was suggested that due to easy access to the internet, both male and female students engaged in various online activities, resulting in no significant difference in loneliness between genders [36]. However, the result contradicted findings from several other studies [8,57]. This inconsistency might be due to differences in sample sizes and/or variations in cultural and social factors. The study found that a student’s place of residence significantly affects their feelings of loneliness. Students from rural areas reported feeling lonelier than those from urban areas. This finding is supported by research from [58,59].

Age played a role, as individuals aged 21 years or younger had higher odds of feeling lonely compared to those older than 21 years. It could have been because it was possibly their first time away from home, family, and friends. Coupled with being in an unfamiliar city and facing new pressures and expectations, it left them understandably feeling lonely, homesick, and struggling with their mental health.

Regarding BMI, both underweight and overweight individuals had significantly higher odds of experiencing loneliness compared to those with normal weight among university students. This results of the study coincided with a recent study. Obesity and loneliness appeared to be connected, negatively affecting both physical and emotional well-being. Some studies argued that malnutrition might increase loneliness [60].

Family type was another significant factor, with individuals from nuclear families having higher odds of feeling lonely compared to those from joint families in this study. This result was similar to other findings [61]. Families played a key role in addressing loneliness. As modern trends shifted from extended to nuclear families, elders often moved to care centers, leaving fewer opportunities for grandchildren to spend time with them [62]. Studies showed that interaction between grandparents and grandchildren helped reduce loneliness and other issues like aggression and depression [61]. Similarly, students from joint families, where multiple generations lived together, had more chances to connect with their grandparents, leading to lower feelings of loneliness.

This study also investigated the importance of family monthly income in the experience of loneliness among university students. This variable was examined because poverty was considered an important factor in loneliness [63]. Children from low-income families might have experienced loneliness due to their restricted participation in social activities and limited social connections [64]. This study found a significant effect of family monthly income, revealing that individuals from lower-income households had higher odds of experiencing loneliness compared to their counterparts. This finding aligned with the study conducted by Özdemir and Tuncay [65]. Finally, the fitted model can correctly predict 76.3% for new respondent.

### Strength and limitation

This was the first time we attempted to investigate loneliness of university students in Bangladesh after COVID-19. We used primary data that was collected directly from students, and they were selected by a suitable statistical technique. A suitable statistical model was used for identifying the predictors of loneliness among students. However, this study had several limitations. Firstly, it employed a cross-sectional design to assess loneliness among university students, capturing only a snapshot of loneliness at one point in time without tracking changes over time. Secondly, data were collected using self-report questionnaires, potentially influenced by social desirability bias, particularly given that participants were studying in a health-related field. However, efforts were made to mitigate this bias by ensuring privacy and informing participants of the survey’s anonymity. Thirdly, the UCLA Loneliness Scale lacks a clear cutoff score to classify individuals as “lonely” or “not lonely,” making it difficult to interpret results consistently across studies [43]. Fourth, since the research was conducted exclusively among students at Rajshahi University, caution should be exercised when generalizing these findings to other universities in Bangladesh. Lastly, we did not consider some important factors of loneliness such as stress, mental health, academic performance. Clearly more researches are required on loneliness among university students in Bangladesh.

## Conclusions

This study found that approximately one-fourth of Rajshahi University students experienced loneliness after the COVID-19 outbreak. Key factors contributing to loneliness included age group, BMI, family monthly income, and type of family. These findings provide valuable insights for targeted intervention strategies. University authorities could establish support programs to offer counseling and mental health services, encourage social interactions, and provide financial assistance or scholarships to students from lower-income families to alleviate financial stress, which can contribute to feelings of loneliness. The government and health organizations should also address these factors to help reduce loneliness among university students.
